# Depressive symptom instability predicts incident mild cognitive impairment and dementia in older adults

**DOI:** 10.1002/alz.71672

**Published:** 2026-07-12

**Authors:** Lauren A. Rutter, Lucas J. Hamilton

**Affiliations:** ^1^ Department of Psychological and Brain Sciences Indiana University Bloomington Indiana USA; ^2^ Indiana Alzheimer's Disease Research Center Indiana University School of Medicine Indianapolis Indiana USA; ^3^ The Irsay Institute Indiana University Bloomington Indiana USA

**Keywords:** Alzheimer's disease, depressive symptoms, longitudinal cohort, symptom instability, trajectory

## Abstract

**INTRODUCTION:**

Neuropsychiatric symptoms frequently precede Alzheimer's disease and related dementias, yet risk prediction models typically rely on baseline depression severity or diagnostic status. We examined whether instability in depressive symptoms predicted incident mild cognitive impairment (MCI) or dementia.

**METHODS:**

Data were drawn from 11,951 older adults enrolled across 42 US Alzheimer's Disease Research Centers and followed for up to 19 years. Depressive symptoms were assessed using the Geriatric Depression Scale and characterized using rule‐based groupings and latent class trajectories. The Structured Life‐Course Modeling Approach (SLCMA) was used to compare four competing temporal hypotheses of risk. Cox models adjusted for demographic factors and apolipoprotein E (APOE) ε4 status.

**RESULTS:**

Fluctuating depressive symptom trajectories were associated with increased risk of incident cognitive impairment. Symptom instability showed the lowest prediction error in exploratory SLCMA analyses.

**DISCUSSION:**

Instability in depressive symptoms may represent an early and clinically accessible risk marker for MCI and dementia.

## Highlights

- Depressive symptom instability predicts incident MCI and dementia.
- Instability showed the lowest predictor error in exploratory SLCMA models.
- Findings replicate across rule‐based and latent class trajectories.

## 1 BACKGROUND

Depression is a common and clinically significant psychiatric condition in later life^1^ and is associated with increased risk of cognitive impairment and dementia. The number of Americans living with Alzheimer's disease and related dementias (ADRD) is projected to double to 13.8 million by 2060, with annual costs reaching US$1 trillion.^2^ In the absence of a cure, studying modifiable risk factors, which account for up to 40% of cases,^3^ is critical to help stave off the public health issues that ADRD poses across the globe. Beyond studies that demonstrate the effects of clinically diagnosed depression and the timing of its onset,^4^ or more recent work that shows how specific symptoms relate to dementia risk,^5^ nearly no studies have examined how instability in depressive symptoms may impact cognition, particularly ADRD risk in older adults. The current work fills this gap testing the prognostic value of instability in depression symptoms using a national sample of over 11,000 US older adults.

Depressive symptoms are among the most common neuropsychiatric symptoms preceding mild cognitive impairment (MCI) and dementia, yet most studies rely on a single‐time‐point severity or diagnostic status. Prior studies of depression and ADRD linked chronically high symptoms with poorer cognitive performance^6^ and faster cognitive decline.^7^, ^8^ Beyond high‐severity cases, very little evidence has been produced that tests whether changes in depression symptoms over time carry additional prognostic value for downstream cognitive health. Variability in depression symptoms can signal a dysregulated stress response to emotion and reduced homeostatic resilience,^9^, ^10^ which are linked to accelerated neurobiological aging.^11^, ^12^ Importantly, mood instability emerges earlier than measurable cognitive changes, with neuropsychiatric symptoms preceding diagnosis in most (55%) people who develop MCI and nearly two‐thirds of cases who develop dementia.^13^ In summary, the emerging literature on depression variability and cognitive decline raises the possibility that instability in depression symptoms can serve as an early warning sign for future ADRD.

**RESEARCH IN CONTEXT**

1. **Systematic review**: We searched PubMed for studies published in English up to February 2026 examining depressive symptoms and subsequent cognitive impairment, using combinations of the terms “depression,” “depressive symptoms,” “late life depression,” “mood instability,” “fluctuations,” “trajectory,” “cognitive decline,” “mild cognitive impairment,” “dementia,” “Alzheimer's disease,” and “ADRD.” Prior research consistently shows that elevated depressive symptoms are associated with increased risk of cognitive decline and dementia. However, most studies operationalize depression using single‐time‐point severity or diagnosis and relatively few models longitudinal symptom trajectories. To our knowledge, no large‐scale study has directly compared competing life‐course hypotheses (accumulation, proximity/recency, critical incident, and instability) in late‐life depression to evaluate which temporal patterns may explain depression‐related cognitive risk.
2. **Interpretation**: Using data from 11,951 older adults across 42 US ADRCs followed for up to 19 years, we examined depressive symptom patterns using both rule‐based (depression cutoff scores) and latent class approaches. We then applied the SLCMA to compare competing temporal hypotheses. In exploratory life‐course comparisons, depressive symptom instability showed the lowest prediction error, although model fit was similar for instability, accumulation, and critical incident hypotheses. These findings suggest that within‐person variability in depressive symptoms may be a more informative risk signal than single‐time‐point symptom severity.
3. **Future directions**: The available evidence suggests that depression is associated with higher risk for cognitive impairment, but our findings indicate that how symptoms change over time may matter more than how severe they are at a single assessment. Repeated assessment of depressive symptom variability may improve early identification of individuals at elevated risk for cognitive impairment and inform more targeted prevention strategies in aging populations.

If depressive symptom instability is associated with increased risk of ADRD, this may be due to any number of plausible mechanisms, including symptom timing (often described in life‐course models as a sensitive period hypothesis, although in the present late‐life design, it is more accurately proximity or recency of symptoms relative to impairment), the severity of any given depressive episode (i.e., critical incident hypothesis), the length of time with elevated symptoms (i.e., accumulated exposure hypothesis), or the extent of symptom fluctuation across time (i.e., instability hypothesis). The latter is the hypothesis being proposed here, but given the lack of literature in this area, the alternative hypotheses remain plausible. To directly compare these competing temporal hypotheses, we applied the Structured Life‐Course Modeling Approach (SLCMA),^14^ which enables direct evaluation of alternative hypotheses including cumulative burden, critical incident, timing/proximity effects, and symptom instability in relation to incident ADRD risk.

This study leverages longitudinal data from the National Alzheimer's Coordinating Center (NACC). Our final analytic dataset includes 11,951 older adults across 42 Alzheimer's Disease Research Centers (ADRCs), comprising over 80,000 observations with standardized assessments of depressive symptoms and ADRD outcomes. This design enables an evaluation of whether temporal instability in depressive symptoms predicts incident ADRD and allows formal comparison of competing life‐course models. To quantify instability (also termed “fluctuations”) in depression over time, this study compared two complementary trajectory phenotypes: (1) a transparent and reproducible rule‐based grouping using pre‐existing clinical cutoff norms and (2) a data‐driven latent class analysis (LCA) that clusters individuals by shared patterns of symptom changes. Fluctuating and persistently high depression symptoms were hypothesized to show higher incident impairment risk compared to low levels of depression given existing evidence.^7^, ^15^ Then, SLCMA^16^ was used to directly compare competing hypotheses, including timing/proximity, critical incident, and accumulated exposure to the instability hypothesis. These approaches are intended to provide complementary perspectives rather than interchangeable operationalizations of instability. While the rule‐based and LCA approaches quantify person‐level depressive symptoms across follow‐up, SLCMA quantifies specific temporal features of depressive symptoms relative to impairment or censoring.

## 2 METHODS

Using the NACC database, data were compiled from 42 ADRCs across the United States, including all participants as of the June 2025 data freeze. The current sample was filtered to include only participants with at least three visits and a minimum follow‐up duration of 5 years to improve model precision (i.e., statistical power, reliable estimates)^17^ and reduce bias associated with sparse follow‐up. See Supplemental Materials for full filtering procedures. The final analytic sample included 11,951 participants (mean age = 73.2 years, 59.5% female) contributing 81,374 visits. The median time to first impairment or censoring was 5.4 years (IQR = 0.0 to 9.1; range = 0.0 to 19.3 years). Across follow‐up, 6576 individuals (55.0%) experienced incident cognitive impairment; the remainder were censored. Incident cognitive impairment was defined as the first observed onset of impairment during longitudinal follow‐up (time to first impairment), including events occurring at a participant's first eligible visit within the analytic sample. See Tables 1A and 1B for sample descriptives.

**TABLE 1a:** Sample descriptives (overall and by rule‐based trajectory).

Type	Group	*N*	Events (*n*)	Events (%)	Age, mean (SD)	Female (%)	Education, mean (SD)	APOE ε4 (%)	Baseline GDS, median [IQR]	Follow‐up years, median [IQR]	Visits, median [IQR]
Overall	Overall	11,951	6576	55.0	73.2 (6.9)	59.5	16.1 (5.7)	36.6	1.0 [0.0 to 2.0]	5.4 [0.0 to 9.1]	6.0 [4.0 to 9.0]
By trajectory	Stable low	8596	4245	49.4	73.1 (6.8)	59.4	16.3 (5.8)	36.5	0.0 [0.0 to 1.0]	6.0 [0.0 to 9.3]	6.0 [5.0 to 9.0]
By trajectory	Stable high	214	179	83.6	73.1 (7.4)	55.6	15.1 (6.9)	39.3	8.0 [6.0 to 10.0]	1.3 [0.0 to 5.1]	4.0 [2.0 to 6.0]
By trajectory	Fluctuating	2665	1871	70.2	73.9 (6.9)	60.2	15.7 (5.2)	35.4	2.0 [1.0 to 5.0]	4.0 [0.0 to 8.6]	7.0 [5.0 to 9.0]

*Note*: Events indicate time‐to‐first impairment. Percentages are within‐group. Participants not classifiable for rule‐based trajectory (*n* = 476; events = 281) are included in the overall row but not shown in trajectory rows. Rule‐based groups used GDS ≥5 as high.

Abbreviations: GDS, Geriatric Depression Scale, IQR, interquartile range.

**TABLE 1b:** Sample descriptives by LCA class.

Group	*N*	Events (*n*)	Events (%)	Age, mean (SD)	Female (%)	Education, mean (SD)	APOE ε4 (%)	Baseline GDS, median [IQR]	Follow‐up years, median [IQR]	Visits, median [IQR]
Low/stable	10,704	5683	53.1	73.3 (6.8)	59.6	16.2 (5.7)	36.1	1.0 [0.0 to 2.0]	5.8 [0.0 to 9.3]	7.0 [5.0 to 9.0]
Moderate/variable	694	545	78.5	73.4 (7.0)	59.2	15.5 (6.7)	39.7	5.0 [4.0 to 7.0]	1.2 [0.0 to 5.7]	5.0 [3.2 to 7.0]
High/stable	77	67	87.0	72.8 (6.7)	54.5	14.4 (3.9)	36.4	11.0 [10.0 to 12.0]	0.0 [0.0 to 4.1]	5.0 [4.0 to 7.0]

*Note*: LCA applied to first 3 visits; classes labeled by increasing symptom severity and stability.

Participants were assessed and diagnosed by in‐house clinicians at each ADRC using the Uniform Data Set (UDS), a collection of measures ranging from demographic questions to neuropsychological tests. Additional data used in the current study include the following variables: age, education, biological sex, ADRC site, the Geriatric Depression Scale (GDS‐15),^18^ APOE ε4 genotyping, diagnostic status, days from first visit, and the total number of all visits. Apart from demographic and genetic variables, which were only collected at the first visit, depressive symptoms were assessed at each visit using the 15‐item GDS, and diagnostic status was assigned at every visit. GDS‐15 scores range from 0 to 15, and higher scores reflect greater depressive symptom burden. Diagnostic status was decided by either a consensus panel or a single physician (i.e., qualified administrator of the neurological exam) across ADRCs.^19^ While a consensus diagnosis from a panel of trained clinicians offers advantages, it is not guaranteed for the entire NACC sample.

### 2.1 Data analysis

All analyses were conducted in R Studio^20^ using the survival, survminer, ggplot2, poLCA, broom, dplyr, tidyr, splines, lars, and selectiveInference packages. To create rule‐based trajectories, visits were coded as high versus low depression using the commonly accepted cutoff of GDS‐15 = 5 to denote a clinical threshold.^21^ This cutoff as opposed to a higher value better characterizes depression in its mild or moderate forms as it commonly emerges in older adults and avoids a biased estimate of ADRD risk by using only the most severely depressed cases.

After coding each visit, individuals were classified as (1) stable low: having no high visits, (2) stable high: no low visits, or (3) fluctuating: one or more high and one or more low visits (Table 1a). Most participants were characterized as stable low (72%), followed by fluctuating (22%), and then stable high (2%). Percentages reflect only participants with sufficient data to derive trajectories and, thus, do not add up to 100%. The stable high group was characterized by persistently elevated GDS symptoms and high rates of conversion (83.6%); however, the fluctuating group had the next highest rates of conversion (70.2%), higher baseline GDS scores, and a shorter follow‐up than the stable low group (49.4% conversion). Cases labeled as fluctuating did not require a specific order (i.e., low to high, high to low).

The LCA used the first three observed visits containing GDS and created ordered indicators based on GDS score (1 = ≤4, 2 = 5 to 9, 3 = ≥10). Fitting with multiple random starts ranging from two to five groups, Bayesian information criteria selected an optimal solution of three groups (see Supplemental Materials for plots depicting model fit across solutions). As shown in Table 1b, these groupings were consistent with characterization as stable low (90% of cases), stable high (6%), and moderate/variable (1%).

Using these grouping variables, Cox proportional‐hazards models were used to examine time to impairment and capture temporally dynamic risks associated with depression trajectories. Results are reported as hazard ratios (HRs) representing the relative likelihood of an event (i.e., conversion) occurring in one group compared to another over time. After constructing baseline models, we assessed proportional hazards with Schoenfeld residuals andб when these were violated, ran time‐varying covariate models using log‐time interactions. Location was included as a frailty term to control unobserved random variability clustered by ADRC site.^22^

To implement SLCMA, we first characterized the temporal pattern of depression relative to cognitive decline by constructing event‐aligned depression exposure windows spanning the 10 years prior to incident impairment or censoring. Visits were aligned backward from the earliest diagnosis or censoring date, and GDS scores within these periods were used to create window‐specific summary indicators. Consistent with the grouping rule, a GDS‐15 score of 5 or greater was coded as elevated depressive symptoms for any given window. Four complementary metrics were derived to represent four distinct life‐course hypotheses: (1) window‐specific indicators (6 to 10 years before impairment event, 3 to 6 years before impairment, and 0 to 3 years before impairment) to evaluate a proximity/recency hypothesis, testing whether elevated depressive symptoms closer to impairment or censoring were especially predictive; (2) proportion of observed windows with elevated symptoms to capture the accumulation hypothesis; (3) rate of transitions between elevated and non‐elevated depressive states across adjacent observed to test the instability hypothesis; and (4) a critical incident indicator, operationalized as any severe depressive episode (GDS ≥ 11) within the event‐aligned windows. Instability was calculated as rate rather than count, and transitions between elevated and non‐elevated depressive symptom states were counted across adjacent window pairs with observed data and divided by the number of observed adjacent window pairs.

To identify which hypothesis best explained variation in time to conversion, SLCMA analyses utilized least angle regression (LARS) using the “lars” package in R Studio. LARS is a variable selection method like the LASSO method that enters each predictor one at a time based on their contribution to model fit. Prior to model fitting, we residualized the continuous outcome proxy (follow‐up time) and exposure variables on covariates using the Frisch‐Waugh‐Lovell procedure. Predictive performance at each step of LARS was evaluated using *k*‐fold cross‐validation. Subsets of participants were iteratively held out for model testing, and cross‐validated mean squared error (CV‐MSE) was calculated to quantify out‐of‐sample‐prediction error across each test. Models with the lowest CV‐MSE were considered the best fit, representing which of the four hypotheses most accurately predicted conversion risk. Because the LARS component of SLCMA performs variable selection using a continuous follow‐up time proxy rather than directly modeling censoring, we interpreted this step as exploratory. Because SLCMA performs variable selection rather than estimation, final effect sizes were obtained from a separate Cox model using the best supported predictor while controlling for age, sex, education, APOE ε4 status, baseline depression, and ADRC location.

## 3 RESULTS

### 3.1 Survival models

**FIGURE 1:**
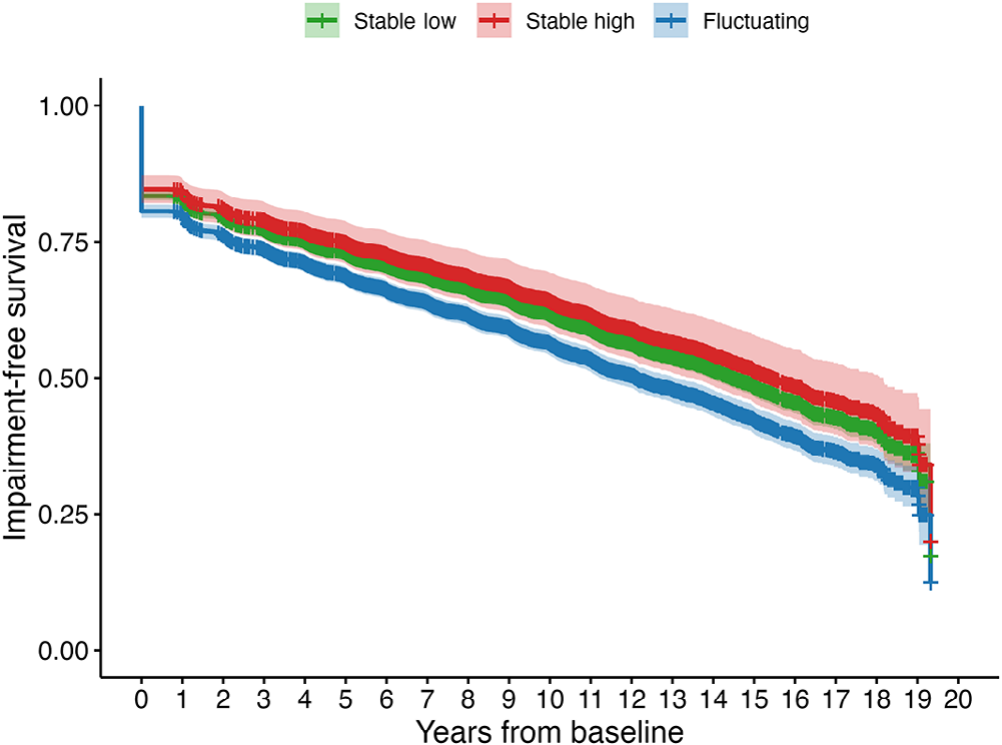
Cox impairment‐free survival by rule‐based trajectories.

Cox proportional‐hazard models were conducted using the three rule‐based depression trajectories and three LCA depression trajectory classes adjusting for age, sex, education, APOE ε4 status, baseline GDS, and ADRC site (frailty term). Using the rule‐based solution, the fluctuating group was predicted to have a higher risk of impairment compared to stable low symptoms (HR = 1.24, 95% confidence interval [CI]: 1.16 to 1.32, *p* < 0.001; Table 2). Covariate‐adjusted survival curves are shown in Figure 1. Unadjusted Kaplan‐Meier curves are provided in Supplemental Materials. Contrary to expectations from an accumulation hypothesis, those in the stable high group were not predicted to have a significantly higher risk (HR = 1.00, 95% CI: 0.84 to 1.20). The model showed good predictive accuracy (C‐index = 0.72, SE = 0.004), and predicted risk accurately distinguished participants who converted earlier or later. When the LCA groups were tested, risk was elevated for the moderate/variable group (HR = 1.08, 95% CI: 0.97 to 1.21) and reduced for the stable high group (HR = 0.78, 95% CI: 0.60 to 1.03), though neither reached significance (Table 2). However, lack of significant effect in the stable high group may represent a subgroup with high conversion rates and limited observations of follow‐up, which we address with a landmark sensitivity analysis, described below. Again, the model showed good predictive accuracy (C‐index = 0.72, SE = 0.004).

**TABLE 2:** Adjusted Cox frailty models.

Model	Group	HR (95% CI)	*p*
LCA (ref = low/stable)	High/stable	0.79 (0.60 to 1.03)	0.081
LCA (ref = low/stable)	Moderate/variable	1.09 (0.98 to 1.21)	0.131
Rule‐based (ref = stable low)	Fluctuating	1.24 (1.16 to 1.32)	<0.001
Rule‐based (ref = stable low)	Stable high	1.00 (0.84 to 1.20)	0.988

*Note*: Models adjust for baseline GDS, age, sex, education, APOE ε4, and ADRC frailty. HRs compare each group to reference shown in model label.

To evaluate whether the predictors in this Cox proportional hazards model satisfied the proportional hazards assumptions, we conducted the Schoenfeld residuals test. For rule‐based models, all predictors except education significantly violated the time‐invariance assumption (all *p*s < 0.001; global *p* = 3.7 × 10^−^^9^). This indicates that HRs are not consistent over time. To address this limitation, we fit time‐varying Cox models that allowed the effects of depression trajectory, age, sex, APOE ε4 status, and baseline depression symptoms to vary as a function of log‐time. The fluctuating and stable high groups had increasing conversion risk over time (Table 3a). In the full sample, the fluctuating group had a 37% higher hazard at 1 year (HR = 1.37, 95% CI: 1.28 to 1.48, *p* < 0.001) and 49% higher hazard at 5 years (HR = 1.49, 95% CI: 1.36 to 1.63, *p* < 0.001), and the stable high group showed a similar pattern, with hazard ratios increasing from 1.41 at 1 year to 1.76 at 5 years. Results were consistent when retesting the time‐varying Cox model stratified to participants who were cognitively normal at baseline (i.e., incident‐only; Table 3b).

**TABLE 3a:** Time‐varying hazards versus stable low (full sample).

Group	Time (years)	HR (95% CI)	*p*
Fluctuating	1	1.37 (1.28 to 1.48)	<0.001
Fluctuating	3	1.45 (1.33 to 1.58)	<0.001
Fluctuating	5	1.49 (1.36 to 1.63)	<0.001
Stable high	1	1.41 (1.13 to 1.75)	0.002
Stable high	3	1.64 (1.27 to 2.11)	<0.001
Stable high	5	1.76 (1.34 to 2.30)	<0.001

*Note*: HR(t) computed with log‐time interaction; *p* values correspond to HR(t) at the specified time.

**TABLE 3b:** Time‐varying hazards versus stable low (incident only).

Group	Time (years)	HR (95% CI)	*p*
Fluctuating	1	1.39 (1.10 to 1.75)	0.005
Fluctuating	3	1.49 (1.32 to 1.68)	<0.001
Fluctuating	5	1.53 (1.39 to 1.69)	<0.001
Stable high	1	1.35 (0.61 to 3.00)	0.457
Stable high	3	1.47 (0.96 to 2.23)	0.075
Stable high	5	1.52 (1.01 to 2.30)	0.046

*Note*: Incident‐only analysis restricts to participants cognitively normal at first visit.

For LCA hazard models, assumptions were not violated; however, for comparison, we ran incident‐only Cox models. Among participants who were cognitively normal at their first visit, the moderate/variable group remained at higher risk of conversion (HR = 1.28, 95% CI: 1.03 to 1.58, *p* = 0.021), while the stable high group did not differ from the stable low reference group (HR = 0.82, 95% CI: 0.42 to 1.60, *p* = 0.56). However, this could be due to a low number of stable high cases, which reduced the power to detect effects, which prompted a landmark sensitivity analysis, described below.

### 3.2 Sensitivity analyses

Importantly, we repeated all preceding analyses with baseline antidepressant use as a control variable. Baseline antidepressant use independently predicted a greater risk of cognitive impairment in the full sample (HR = 1.49, *p* < 0.001) and incident‐only subset (HR = 1.31, *p* < 0.001). However, adjustment for antidepressant use did not meaningfully alter the primary rule‐based trajectory or LCA findings: the fluctuating group remained associated with increased impairment risk in both the full sample (HR = 1.20, *p* < 0.001) and incident‐only subset (HR = 1.50, *p* < 0.001), and the moderate/variable LCA class remained associated with increased risk in the incident‐only subset (HR = 1.26, *p* = 0.034).

In a 2‐year landmark sensitivity analysis excluding participants who converted to impairment within 2 years of baseline, both the stable high (HR = 1.89, 95% CI: 1.39 to 2.56, *p* < 0.001) and fluctuating (HR = 1.48, 95% CI: 1.34 to 1.64, *p* < 0.001) groups were associated with increased risk of impairment relative to the stable low group. Thus, the stable high estimate from the original models above should be interpreted with caution as the results may reflect early conversion and limited observed at‐risk time.

In sex‐stratified sensitivity analyses, the fluctuating trajectory remained significantly associated with incident impairment in both males (HR = 1.21, 95% CI: 1.10 to 1.33, *p* < 0.001) and females (HR = 1.25, 95% CI: 1.14 to 1.36, *p* < 0.001). Moreover, the fluctuating x sex interaction was not statistically significant (HR = 1.10, *p* = 0.091), suggesting that the fluctuating trajectory association was not driven by one sex.

### 3.3 Structured Life‐Course Modeling Approach

Using SLCMA to compare competing hypotheses linking depression exposure to cognitive impairment, the instability hypothesis showed the lowest cross‐validated mean squared error, although the model fit was very similar for the instability, accumulation, and critical incident hypotheses. The CV‐MSE values were 19.76 for instability, 19.81 for accumulation, 19.81 for critical incident, and 25.24 for proximity/recency. This pattern provides modest support for the instability hypothesis, although the small differences in CV‐MSE indicate that instability, accumulation, and critical incident models performed similarly in the exploratory model‐selection step. In the confirmatory Cox proportional‐hazards model adjusting for age, sex, education, APOE ε4 carrier status, baseline GDS score, and ADRC location, higher instability in depression symptoms (i.e., greater proportion of switches between elevated and non‐elevated depressive symptom states) was associated with greater hazard of conversion (HR = 1.44; 95% CI: 1.21 to 1.70, *p* < 0.001). In a sensitivity analysis adding the total number of visits contributing to each participant's event‐aligned SLCMA windows, the instability association was weakened, but it remained significant (HR = 1.29, 95% CI: 1.10 to 1.53, *p* = 0.002).

## 4 DISCUSSION

In support of the instability hypothesis, fluctuations in depression symptoms consistently predicted increased risk of conversion to ADRD across all analyses. The fluctuating groups carried the highest risk for conversion to AD, and the SLCMA analysis provided modest support for instability, which showed the lowest CV‐MSE. Importantly, these analyses capture complementary but distinct aspects of depressive symptom change. While the rule‐based and LCA classes identify individuals with changes in states over their entire longitudinal follow‐up, SLCMA captures the rate of transitions between elevated and non‐elevated depressive states within event‐aligned windows. Effects emerged after accounting for baseline depression severity, antidepressant use, age, sex, education, and genetic risk, which suggests that instability in depression symptoms may be an early indicator of neurodegeneration.

These findings demonstrate how the trajectory of depression symptoms across time can carry prognostic value.^23^, ^24^, ^25^ One possibility is that fluctuating depressive symptoms reflect repeated affective dysregulation and activated physiological stress, which reinforce each other over time. This pattern may contribute to allostatic load and increase vulnerability to decline through multiple pathways (neuroendocrine, inflammatory, cerebrovascular).^26^, ^27^, ^28^ Meta‐analytic data suggest that treating depression could prevent up to 11% of ADRD cases,^29^ with persistent depression being particularly harmful due to chronic exposure to stress hormones, neuroinflammation, and reduced neuroplasticity.^30^ However, fluctuations in depression may reflect impaired regulatory symptoms and global vulnerability to neurodegeneration.^9^, ^10^ Within‐person mood variability is linked to heightened allostatic load, lower psychological wellbeing, and cognitive inefficiency.^31^, ^32^

It is notable that the instability/fluctuation hypothesis showed the lowest prediction error in the SLCMA analyses, given that the critical incident hypothesis is better represented in the literature.^33^ Severe depressive episodes have been linked with hippocampal atrophy and heightened dementia risk, suggesting that a critical episode may matter more.^34^ Similarly, the cumulative burden of chronic depression is known to produce wear and tear on the brain and body over time.^25^ However, these results suggest that instability in depressive symptoms may capture a risk‐relevant pattern^35^ that may be missed by more traditional views of depression severity or chronicity.

These results, along with existing work,^7^ suggest that repeated measures of depression, even brief yearly screens, carry prognostic value beyond baseline depression scores. More specifically, repeated brief monitoring can help understand risk, and plotting trajectories can guide monitoring for individualized prevention efforts. Of note, while the fluctuating course of depression was associated with the highest hazard for incident impairment, our study did not test whether a momentary fluctuating state increased risk directly. Instead, we examined long‐term, between‐visit changes in depressive symptoms over years of follow‐up. Ecological momentary assessment approaches to depression fluctuation and cognitive risk are a potential extension of this research, as it could allow more nuanced and granular detection of risk. The NACC data contain yearly measurements, but daily, weekly, or even monthly assessments could provide more sensitive data on fluctuation patterns in older adults and possibly more accurate risk predictions.

A key strength of the current study is our longitudinal approach into temporal dynamics of depression and ADRD onset. By assessing time to event through Cox proportional hazards models, depression was shown to influence ADRD risk at baseline but increase over time, particularly if it followed a fluctuating course. Prior work showed that depression had a robust, significantly greater impact on women, accelerating risk over time, but did not previously account for the temporal course of depression symptoms.^36^ Moreover, the inclusion of APOE *ε*4 in our analysis not only benchmarks the relative risk presented by depression against other risk factors but also offers insights into potential shared mechanisms, such as neuroinflammation or vascular dysfunction, which were not the target of this study. Future research could leverage these findings to untangle the complicated depression–ADRD relationship by considering additional mediators, such as caregiving stress or hormonal factors, that may clarify causal pathways.

Despite the strengths of this study, several limitations must be considered. First, our stable high LCA class was very small, rendering misclassification possible. Class assignment was done without a formal three‐step or Bolck‐Croon‐Hagenaars correction for classification error. However, given high entropy (0.92), the likelihood of classification error is small in our study. Second, rule‐based groupings relied on a GDS threshold of 5 to define elevated depression symptoms. Although this cutoff is widely used, it provides a simplified representation of severity and yielded a small stable high group. Importantly, LCA findings were consistent with rule‐based results, suggesting the observed relationships are not an artifact of the GDS threshold.

While the NACC dataset is a major asset given its longitudinal assessment of older adults with depression assessments at annual follow‐ups, we could not test sensitive‐period effects in the traditional developmental sense, which would require exposure information from early‐life or midlife events. Instead, our SLCMA windows were aligned backward from impairment or censoring. Many individuals in the sample did not have long pre‐conversion histories. Consequently, in SLCMA analyses, sparse data in earlier windows (i.e., middle or younger adulthood) limited the ability to truly test sensitive‐period effect, and instead we approximated proximity/recency effects.

Additional aspects of SLCMA analysis that warrant caution. In calculating a metric of instability to test the instability hypothesis using rate of transitions across adjacent windows, the number of visits within a window is related to the number of opportunities to be classified as elevated. We directly addressed this with a sensitivity analysis that controlled for the number of visits. Finally, the LARS selection step of the SLCMA approach used revisualized follow‐up time as a continuous outcome proxy rather than directly modeling censoring with time‐to‐event data. We therefore interpreted the LARS step as an exploratory model‐selection step, and final interpretations were based on the Cox models. These constraints should be considered when interpreting results.

An additional limitation of the NACC dataset is its unusual depression patterns across all observations. The NACC dataset contains more depressed males than females, inconsistent with rates of depression in nationally representative epidemiological studies.^37^ While sex differences were not the focus of this paper, it is possible that the selection into NACC contains an oversample of subpopulations that are disproportionately male, likely to be depressed, and at higher ADRD risk than the general population, which may introduce bias in the NACC dataset and limit generalizability. This sampling issue is a limitation of all longitudinal studies and does not invalidate the present findings. In this study, sex‐stratified sensitivity analyses showed a significant association between fluctuating depressive symptoms and incident impairment in both males and females. While the NACC dataset provides invaluable insights, addressing its demographic limitations could further enhance its utility, which will be made possible by UDSv4.0's emphasis on social determinants of health to capture a more representative picture of ADRD risk in diverse populations.

Future research should examine how daily or weekly fluctuations in depressive symptoms relate to cognitive performance and subsequent cognitive decline. A consideration for future study is utilizing high‐throughput designs such as ecological momentary assessment and digital phenotyping, which offer tools for capturing affective instability and cognitive change in real time. However, deploying them in older adults poses both practical and methodological challenges, including participant burden, technology barriers, and attrition.^38^, ^39^, ^40^, ^41^ Additional longitudinal work is needed to determine whether targeting affective instability can reduce ADRD risk. Interventions such as emotion regulation, mindfulness, and cognitive behavioral therapies show efficacy in stabilizing affect and reducing relapse in mood disorders,^42^ but their effect on older adults and cognitive outcomes is largely unknown. Future studies should consider recruiting older adults with intermittent mild to moderate depressive symptoms and include a broader range of cognitive profiles as most research to date has focused on depression diagnostic status rather than symptom patterns. Moreover, much of the existing work has studied depression in already at‐risk older adults. Understanding whether mood symptom instability is a modifiable preclinical mechanism to neurodegeneration could meaningfully hone prevention efforts.

In sum, fluctuations in depressive symptoms predicted incident impairment beyond baseline depression severity and remained robust across analytic approaches and sensitivity analyses. These results suggest that dynamic patterns of mood symptoms may indicate vulnerability to cognitive decline and could offer a target for early intervention. Future work that integrates repeated measures of affect, cognition, and biomarkers is important for clarifying the mechanisms that link mood symptoms and ADRD risk.

## CONFLICT OF INTEREST STATEMENT

The authors declare no conflicts of interest. Author disclosures are available in the Supporting Information.

## CONSENT STATEMENT

All human subjects provided informed consent.

## Supporting information



Supporting information

Supporting information

## References

[1] Zenebe Y , Akele B , W/Selassie M , Necho M . Prevalence and determinants of depression among old age: a systematic review and meta‐analysis. Ann Gen Psychiatry. 2021;20: 55. doi:10.1186/S12991-021-00375-X 34922595 PMC8684627

[2] 2023 Alzheimer's disease facts and figures. Alzheimers Dement 2023;19:1598‐1695. doi:10.1002/ALZ.13016 36918389

[3] Livingston G , Huntley J , Sommerlad A , et al. Dementia prevention, intervention, and care: 2020 report of the Lancet Commission. The Lancet. 2020;396:413‐446. doi:10.1016/S0140-6736(20)30367-6/ASSET/2059B509-1487-4490-AD60-C684EA3CD4C9/MAIN.ASSETS/GR4.JPG PMC7392084 32738937

[4] Ownby RL , Crocco E , Acevedo A , John V , Loewenstein D . Depression and risk for Alzheimer disease: systematic review, meta‐analysis, and metaregression analysis. Arch Gen Psychiatry. 2006;63:530‐538. doi:10.1001/ARCHPSYC.63.5.530 16651510 PMC3530614

[5] Frank P , Singh‐Manoux A , Pentti J , et al. Articles specific midlife depressive symptoms and long‐term dementia risk: a 23‐year UK prospective cohort study. Lancet Psychiatry. 2026;13:100‐111. doi:10.1016/S2215-0366(25)00331-1 41412145

[6] Dintica CS , Habes M , Schreiner PJ , Launer LJ , Yaffe K . Trajectories in depressive symptoms and midlife brain health. Transl Psychiatry. 2024;14:1‐7. doi:10.1038/s41398-024-02883-2 38553474 PMC10980805

[7] Kaup AR , Byers AL , Falvey C , et al. Trajectories of depressive symptoms in older adults and risk of dementia. JAMA Psychiatry. 2016;73:525‐531. doi:10.1001/JAMAPSYCHIATRY.2016.0004 26982217 PMC5082978

[8] Formánek T , Csajbók Z , Wolfová K , et al. Trajectories of depressive symptoms and associated patterns of cognitive decline. Sci Rep. 2020;10:1‐11. doi:10.1038/s41598-020-77866-6 33257789 PMC7705007

[9] Stawski RS , Sliwinski MJ , Hofer SM . Between‐person and within‐person associations among processing speed, attention switching, and working memory in Younger and Older Adults. Exp Aging Res. 2013;39:194‐214. doi:10.1080/0361073X.2013.761556 23421639 PMC3622283

[10] Sliwinski MJ , Smyth JM , Hofer SM , Stawski RS . Intraindividual coupling of daily stress and cognition. Psychol Aging. 2006;21:545‐557. doi:10.1037/0882-7974.21.3.545 16953716 PMC2879634

[11] Rutter LA , Vahia IV , Passell E , Forester BP , Germine L . The role of intraindividual cognitive variability in posttraumatic stress syndromes and cognitive aging: a literature search and proposed research agenda. Int Psychogeriatr. 2021;33:677‐687. doi:10.1017/S1041610220000228 32172714

[12] Rutter LA , Hamilton LJ . Cognitive dispersion and depressive symptoms in aging: distinct contributions to longitudinal decline. J Gerontol B Psychol Sci Soc Sci. 2026;81:gbag055. doi:10.1093/geronb/gbag055 41934111 PMC13273408

[13] Wise EA , Rosenberg PB , Lyketsos CG , Leoutsakos JM . Time course of neuropsychiatric symptoms and cognitive diagnosis in National Alzheimer's Coordinating Centers volunteers. Alzheimers Dement. 2019;11:333‐339. doi:10.1016/J.DADM.2019.02.006 PMC6476801 31024987

[14] Smith ADAC , Hardy R , Heron J , et al. A structured approach to hypotheses involving continuous exposures over the life course. Int J Epidemiol. 2016;45:1271‐1279. doi:10.1093/IJE/DYW164 27371628 PMC5841633

[15] Jones DR , Ruiz JM , Schreier HMC , et al. Mean affect and affect variability may interact to predict inflammation. Brain Behav Immun. 2023;109:168‐174. doi:10.1016/J.BBI.2023.01.008 36681360 PMC10023429

[16] Ben‐Shlomo Y , Kuh D . A life course approach to chronic disease epidemiology: conceptual models, empirical challenges and interdisciplinary perspectives. Int J Epidemiol. 2002;31:285‐293. doi:10.1093/IJE/31.2.285 11980781

[17] Austin PC . Statistical power to detect violation of the proportional hazards assumption when using the Cox regression model. J Stat Comput Simul. 2018;88:533‐552. doi:10.1080/00949655.2017.1397151 29321694 PMC5758343

[18] Yesavage JA , Brink TL , Rose TL , et al. Development and validation of a geriatric depression screening scale: a preliminary report. J Psychiatr Res. 1982;17:37‐49. doi:10.1016/0022-3956(82)90033-4 7183759

[19] Besser L , Kukull W , Knopman DS , Chui H , Galasko D , Weintraub S , et al. Version 3 of the National Alzheimer's Coordinating Center's uniform data set. Alzheimer Dis Assoc Disord. 2018;32:351‐358. doi:10.1097/WAD.0000000000000279 30376508 PMC6249084

[20] RStudio.Team RStudio integrated development for R. RStudio, PBC, Boston. 2020. References—Scientific Research Publishing n.d.https://www.scirp.org/reference/referencespapers?referenceid=3542223(accessed September 20, 2025)

[21] Yesavage JA . The use of self‐rating depression scales in the elderly. In: Handbook for Clinical Memory Assessment of Older Adults American Psychological Association; 2004:213‐217. doi:10.1037/10057‐017

[22] Therneau TM . Survival Analysis [R package survival version 3.8‐3]. CRAN: Contributed Packages 2024. doi:10.32614/CRAN.PACKAGE.SURVIVAL

[23] Yin J , John A , Cadar D . Bidirectional associations of depressive symptoms and cognitive function over time. JAMA Netw Open. 2024;7:e2416305. doi:10.1001/JAMANETWORKOPEN.2024.16305 38861255 10.1001/jamanetworkopen.2024.16305 PMC11167501

[24] Kuchibhatla MN , Fillenbaum GG , Hybels CF , Blazer DG . Trajectory classes of depressive symptoms in a community sample of older adults. Acta Psychiatr Scand. 2011;125:492‐501. doi:10.1111/J.1600‐0447.2011.01801.X 22118370 10.1111/j.1600-0447.2011.01801.x PMC3539152

[25] Mirza SS , Wolters FJ , Swanson SA , et al. 10‐year trajectories of depressive symptoms and risk of dementia: a population‐based study. Lancet Psychiatry. 2016;3:628‐635. doi:10.1016/S2215‐0366(16)00097‐3 27138970 10.1016/S2215-0366(16)00097-3

[26] Harrison NA , Brydon L , Walker C , Gray MA , Steptoe A , Critchley HD . Inflammation causes mood changes through alterations in subgenual cingulate activity and mesolimbic connectivity. Biol Psychiatry. 2009;66:407‐414. doi:10.1016/J.BIOPSYCH.2009.03.015 19423079 10.1016/j.biopsych.2009.03.015 PMC2885494

[27] Mac Giollabhui N , Ng TH , Ellman LM , Alloy LB . The longitudinal associations of inflammatory biomarkers and depression revisited: systematic review, meta‐analysis, and meta‐regression. Mol Psychiatry. 2021;26:3302‐3314. doi:10.1038/S41380‐020‐00867‐4 32807846 10.1038/s41380-020-00867-4 PMC7887136

[28] Danese A , McEwen BS . Adverse childhood experiences, allostasis, allostatic load, and age‐related disease. Physiol Behav. 2012;106:29‐39. doi:10.1016/j.physbeh.2011.08.019 21888923 10.1016/j.physbeh.2011.08.019

[29] Norton S , Matthews FE , Barnes DE , Yaffe K , Brayne C . Potential for primary prevention of Alzheimer's disease: an analysis of population‐based data. Lancet Neurol. 2014;13:788‐794. doi:10.1016/S1474‐4422(14)70136‐X 25030513 10.1016/S1474-4422(14)70136-X

[30] Diniz BS , Teixeira AL , Machado‐Vieira R , et al. Reduced cerebrospinal fluid levels of brain‐derived neurotrophic factor is associated with cognitive impairment in late‐life major depression. J Gerontol B Psychol Sci Soc Sci. 2014;69:845‐851. doi:10.1093/GERONB/GBU096 25149921 10.1093/geronb/gbu096 PMC4217554

[31] Stawski RS , Mogle J , Sliwinski MJ . Intraindividual coupling of daily stressors and cognitive interference in old age. J Gerontol B Psychol Sci Soc Sci. 2011;66(suppl1):i121‐i129. doi:10.1093/GERONB/GBR012 21743045 10.1093/geronb/gbr012 PMC3132765

[32] Dork J , Mangan E , Burns L , Dimenstein E . Affective instability: impact of fluctuating emotions on regulation and psychological well‐being. Behav Sci. 2024;14:783. doi:10.3390/BS14090783 39335997 10.3390/bs14090783 PMC11429290

[33] Sheline YI , Wang PW , Gado MH , Csernansky JG , Vannier MW . Hippocampal atrophy in recurrent major depression. Proc Natl Acad Sci U S A. 1996;93:3908‐3913. doi:10.1073/PNAS.93.9.3908 8632988 10.1073/pnas.93.9.3908 PMC39458

[34] Munro CE , Farrell M , Hanseeuw B , et al. Change in Depressive Symptoms and Longitudinal Regional Amyloid Accumulation in Unimpaired Older Adults. JAMA Netw Open. 2024;7:e2427248. doi:10.1001/JAMANETWORKOPEN.2024.27248 39207757 10.1001/jamanetworkopen.2024.27248 PMC11362871

[35] Bos EH , de Jonge P , Cox RFA . Affective variability in depression: revisiting the inertia–instability paradox. Br J Psychol. 2018;110:814. doi:10.1111/BJOP.12372 30588616 10.1111/bjop.12372 PMC6899922

[36] Rutter LA , Hamilton LJ , Wilson S , Krendl AC , Perry BL . Sex modifies the relationship between depression and risk for dementia: implications for targeted prevention. World Psychiatry. 2026;25:154‐155. doi:10.1002/WPS.70022 41536108 10.1002/wps.70022 PMC12805041

[37] Kessler RC , Berglund P , Demler O , et al. The epidemiology of major depressive disorder: results from the National Comorbidity Survey Replication (NCS‐R). JAMA. 2003;289:3095‐3105. doi:10.1001/JAMA.289.23.3095 12813115 10.1001/jama.289.23.3095

[38] Moore RC , Kaufmann CN , Rooney AS , et al. Feasibility and acceptability of ecological momentary assessment of daily functioning among older adults with HIV. Am J Geriatr Psychiatry. 2017;25:829‐840. doi:10.1016/J.JAGP.2016.11.019 28065496 10.1016/j.jagp.2016.11.019 PMC5453849

[39] Singh S , Strong R , Xu I , et al. Ecological Momentary Assessment of Cognition in Clinical and Community Samples: reliability and Validity Study. J Med Internet Res. 2023;25:e45028. doi:10.2196/45028 37266996 10.2196/45028 PMC10276323

[40] Hakun JG , Roque NA , Gerver CR , Cerino ES . Ultra‐brief assessment of working memory capacity: ambulatory assessment study using smartphones. JMIR Form Res. 2023;7:e40188. doi:10.2196/40188 36705953 10.2196/40188 PMC9919550

[41] Cerino ES , Katz MJ , Wang C , et al. Variability in cognitive performance on mobile devices is sensitive to mild cognitive impairment: results from the Einstein aging study. Front Digit Health. 2021;3:758031. doi:10.3389/FDGTH.2021.758031 34927132 10.3389/fdgth.2021.758031 PMC8677835

[42] Shapero BG , Abramson LY , Alloy LB . Emotional reactivity and internalizing symptoms: moderating role of emotion regulation. Cognit Ther Res. 2015;40:328‐340. doi:10.1007/S10608‐015‐9722‐4 10.1007/s10608-015-9722-4 PMC4876867 27231404

