# Supplementary material for: Depressive symptom instability predicts incident mild cognitive impairment and dementia in older adults
Source: Alzheimers Dement. 2026 Jul 12;22(7):e71672. doi: 10.1002/alz.71672 (PMC13357699; doi:10.1002/alz.71672)

**Supplemental Information**

**Data Cleaning and Filtering:** Listwise deletion was used to exclude participants that did not meet the criteria for each data filtering step. First, participants younger than 65 were excluded from analyses due to a distinct etiology of highly heritable, monogenic early-onset ADRD that differ etiologically from the more common late-onset, multifactorial form of ADRD. Next, participants were categorized to indicate cognitive status (0 = normal cognition, 1 = mild cognitive impairment or ADRD). Importantly, the NACC dataset includes cases that were cognitively impaired but not related to ADRD which were excluded from these analyses. Additionally, APOE *ε*4 genotype was binarized to indicate presence of at least one *ε*4 allele (0 = none, 1 = at least one).

**Table S1. *Sample Filtering flow from raw data file to final analytic dataset***

| Step | Participants | Visits |
| --- | --- | --- |
| Raw NACC file (June 2025 data freeze) | 54,631 | 204,031 |
| Age ≥ 65 years | 45,299 | 172,609 |
| Exclude NACCUDSD = 2 (impaired, not MCI) | 44,458 | 165,047 |
| Known race (exclude RACE = 99) | 44,171 | 164,413 |
| Known APOE ε4 status (exclude NACCNE4S = 9) | 33,764 | 143,672 |
| Valid GDS (exclude NACCGDS = -4 or 88) | 32,142 | 125,700 |
| Non-zero follow-up days (exclude NACCDAYS = 0) | 26,298 | 119,856 |
| Eligibility: ≥3 visits and ≥5 years observed follow-up | 11,952 | 81,493 |
| Final analytic dataset (after survival construction and modeling restrictions) | 11,951 | 81,374 |
| *Note.* Participants = unique IDs; Visits = total rows at each step; NACCUDSD = National Alzheimer’s Coordinating Center Uniform Dataset Diagnosis; GDS = Geriatric Depression Scale; The eligibility row reflects prespecified inclusion criteria applied to the NACC dataset. The final analytic dataset may differ slightly due to survival dataset construction and complete-case requirements for modeling. | | |

**LCA fit indices**

**Figure S1. *Model fit indices for Latent Class Analysis.*** *
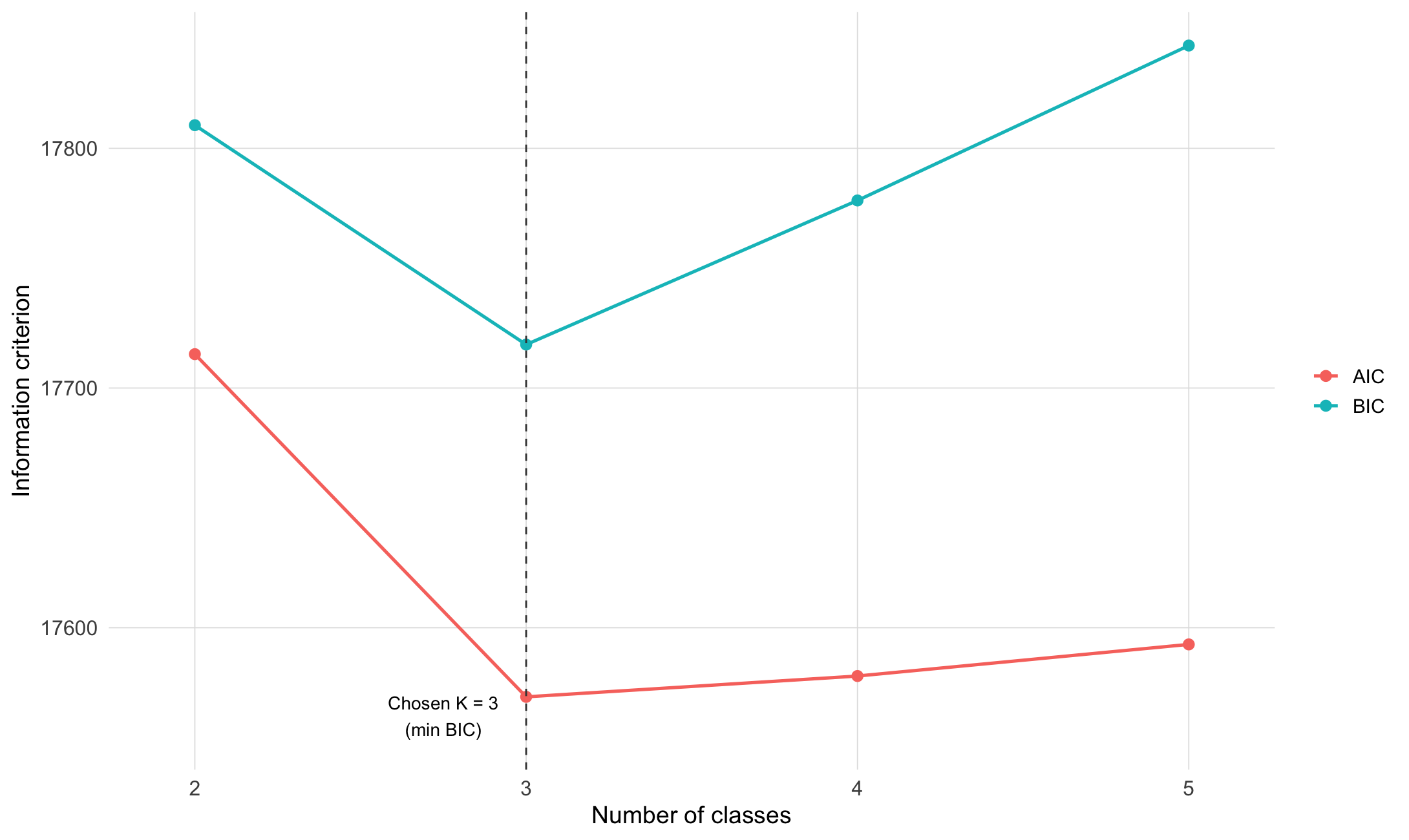
Note.* Model selection for latent class analysis using Akaike information criterion (AIC) and Bayesian information criterion (BIC) across 2-5 class solutions. The 3-class solution minimized the BIC and was selected as the optimal balance between model fit and parsimony. Posterior classification quality was high for the selected 3-class solution, with an average posterior probability of assigned class membership of .971 and relative entropy of .922.

**Table S2.** ***LCA model selection***

| **Number of classes** | **AIC** | **BIC** |
| --- | --- | --- |
| 2 | 17714.127 | 17809.65 |
| 3 | 17571.1608 | 17718.1193 |
| 4 | 17579.8485 | 17778.2425 |
| 5 | 17593.0429 | 17842.8724 |

**Figure S2. *Kaplan-Meier impairment-free survival curves by rule-based and LCA trajectories***


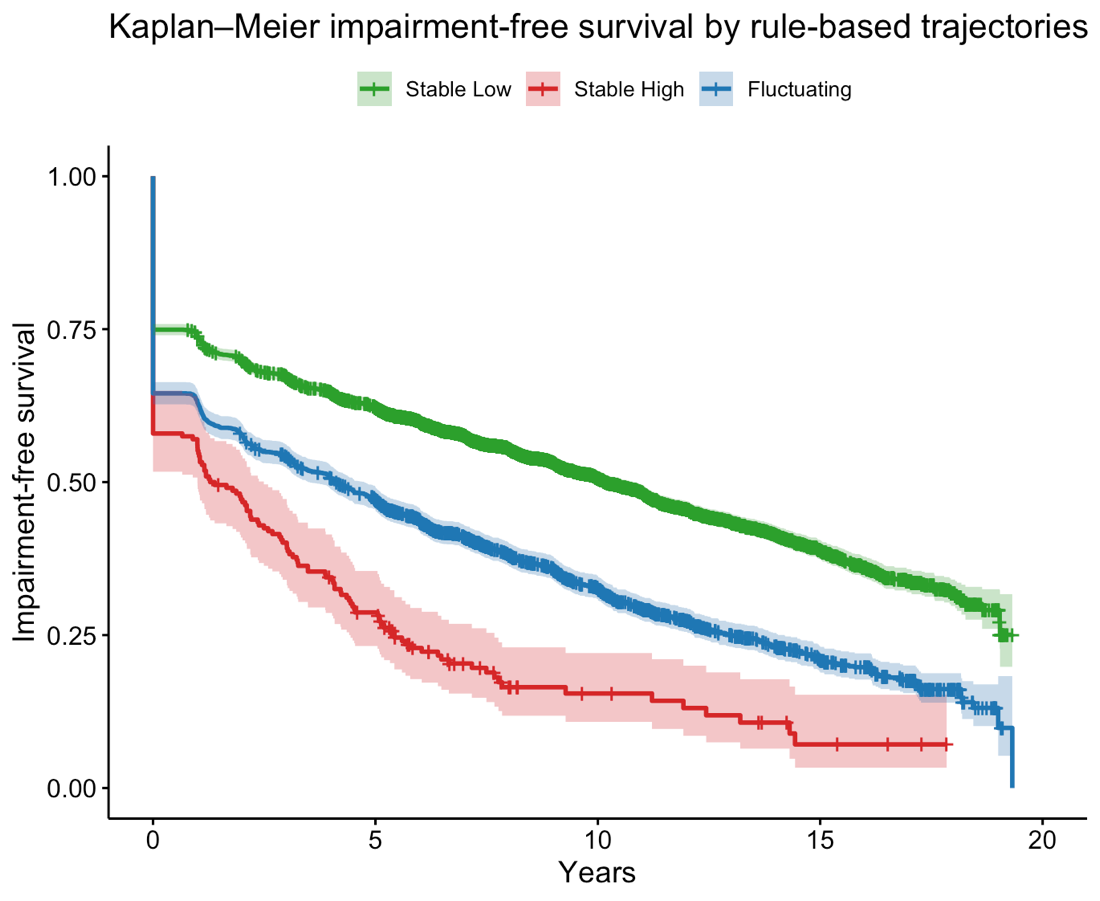

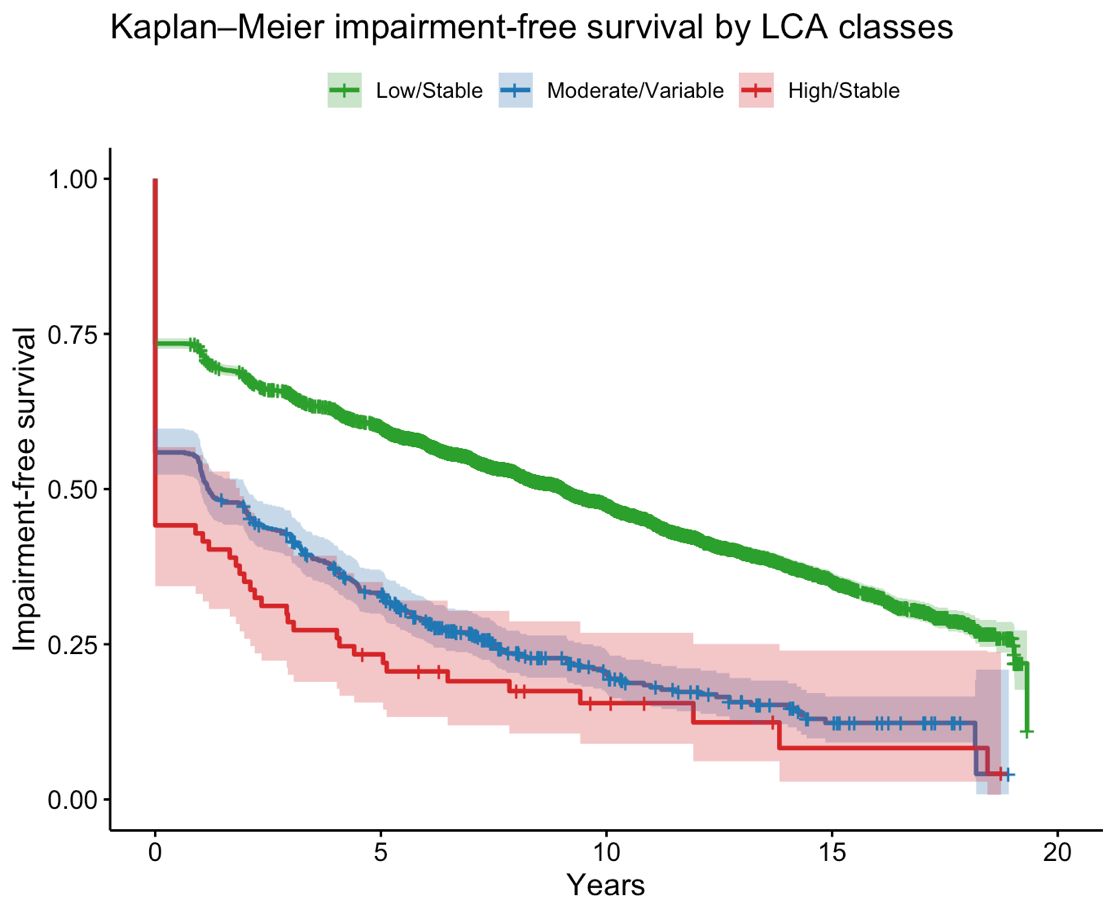

Supplement: Supplementary file 1 — Supporting information [file ALZ-22-e71672-s001.docx]
